# The evaluation of preventive and control measures on congenital syphilis in Guangdong Province, China: a time series modeling study

**DOI:** 10.1007/s15010-022-01791-1

**Published:** 2022-03-17

**Authors:** XiJia Tang, Wen Chen, Shang Qing Tang, Pei Zhen Zhao, Li Ling, Cheng Wang

**Affiliations:** 1 School of Public Health, Sun Yat-Sen University, Guangzhou, 510080 Guangdong China; 2 Dermatology Hospital, Southern Medical University, Guangzhou, 510091 Guangdong China; 3 Institute for Global Health and Sexually Transmitted Disease, Southern Medical University, Guangzhou, 510091 Guangdong China; 4 Sun-Yat Sen University Cancer Center, Guangzhou, 510080 Guangdong China

**Keywords:** Congenital syphilis, Health policy, Interrupted time series analysis, ARIMA

## Abstract

**Purpose:**

To evaluate the effectiveness of preventive and control measures for congenital syphilis (CS) implemented since 2012 in Guangdong Province, China, and assess the epidemic trend in the near future.

**Methods:**

The interrupted time series analysis was conducted to compare changes in slope and level of CS notification rate from 2005 to 2020 in Guangdong Province and its three regions with different economic developmental levels. The ARIMA model was established to predict the new CS case number of Guangdong Province in 2021.

**Results:**

A total of 12,687 CS cases were reported from 2005 to 2020. The CS notification rate of the province had been increasing until 2012 (128.55 cases per 100,000 live births) and then been decreasing constantly, hitting the lowest point in 2020 (5.76 cases per 100,000 live births). The severe epidemic cluster shifted from the developed region to underdeveloped ones over time. The effectiveness of the measures was proved by the significant change in the slope of the notification rate which was found in both of the provinces (− 18.18, 95% CI − 25.63 to − 10.75) and two less-developed regions (− 10.49, 95% CI − 13.13 to − 7.86 and − 32.89, 95% CI − 41.67 to − 24.10, respectively). In the developed region where the notification rate had already been decreasing in the pre-implementation period, implementing these measures also aided in hastening the rate of descent. The CS case number in 2021 was predicted to be 48, indicating a low-level epidemic.

**Conclusions:**

The preventive and control measures have assisted Guangdong Province to control CS effectively, of which the supportive ones ensured a successful implementation. For resource-limited countries where CS is still endemic, especially guaranteeing the support in financial subsidy, professional training, supervision and so on might trigger the effectiveness of other measures and eventually make significant and sustainable progress.

**Supplementary Information:**

The online version contains supplementary material available at 10.1007/s15010-022-01791-1.

## Background

Congenital syphilis (CS) is a type of syphilis that can be transmitted from mother to child during pregnancy and cause adverse birth outcomes (ABO) such as fetal anemia, stillbirth or even perinatal death in up to 80% of CS cases [1, 2]. It has re-emerged worldwide over the last few decades and become an important contributor to infant morbidity and mortality [3]. More than 500,000 cases of CS were reported globally in 2016, resulting in over 200,000 stillbirths and neonatal deaths, according to the World Health Organization (WHO) [4]. Fortunately, this disease can be effectively prevented and eventually eliminated through a variety of cost-effective interventions, such as early screening in antenatal care and immediate treatment [4]. Eliminating CS could benefit not only in reducing ABO attributable to syphilis, but also in improving maternal health, contributing to the achievement of the Sustainable Development Goals (SDG) of the United Nations.[5]. WHO therefore initiated a series of programs, such as the *Global health sector strategy on sexually transmitted infections 2016–2021*.

China has also experienced the resurgence since the 1990s. The notification rate of CS jumped from 0.53 cases per 100,000 live births in 1997–64.41 cases per 100,000 live births in 2009, with an annual increase of 49.2% [6]. To respond to the rising epidemic, China piloted “Regulations for the prevention of mother-to-child transmission of AIDS, syphilis and hepatitis B” in 2010 [7], as well as “The National Program of Syphilis Control and Prevention 2010–2020” [6]. Both of the policies set controlling CS notification rate under 30 cases per 100,000 live births by 2015 as the mid-term benchmark and 15 cases per 100,000 live births by 2025 as the final goal for successfully controlling CS. What they had in common were the CS preventive and control measures, specifically:

(a) Integrating the health education or consultation with antenatal care for pregnant women and encouraging their partners to get tested. The syphilis screening service in Guangdong is free for pregnant women at their first antenatal visit [8]. If they are tested positive, their partners will be encouraged to get tested for free as well.

(b) Implementing prevention of mother-to-child transmission (PMTCT) services (including syphilis screening and treatment) for the infected mothers and infants as early as possible. The test for diagnosing syphilis contains the treponemal tests (such as *Treponema pallidum* particle agglutination, TPPA) and nontreponemal ones (such as venereal disease research laboratory, VDRL). If the pregnant woman was diagnosed with syphilis, she would be treated with is benzathine penicillin 2.4 million units IM in a single dose per week for 3 weeks. Monthly nontreponemal test is required after the treatment [9].

(c) Standardizing the treatment and follow-up management for infants who tested positive for the antibody of syphilis. For example, for those whose mother received regular treatment during pregnancy, infants with reactive nontreponemal and treponemal test results after birth, and the nontreponemal antibody titers fewer than fourfold of its mother’s, should receive regular serologic tests every 3 months until the test becomes nonreactive; otherwise, it should receive standard treatment. Infants with nonreactive test results should be retested at the 1st, 2nd, 3rd and 6th month after birth. If the result is still nonreactive till the 6th month, no further evaluation is needed [9].

(d) Providing supportive measures by the government, such as financial subsidy, training for health workers and regular supervision.

The first three measures can be considered as evidence-based interventions, as several countries had been validated as having eliminated CS by adopting them, such as Thailand and Cuba [10, 11]. They were also addressed in the national syphilis control guideline of some developed countries, such as New Zealand and England [12, 13]. Besides, the supportive measures, including the supervision and data management, the sufficient financial subsidy for each aspect of the implementation, and the capacity building of health professionals (such as the training of maternal and child health-care professionals and the improvement of infrastructure and laboratories of primary health-care institutions), were another important component to ensure the effectiveness of other CS control measures, which was also emphasized by WHO [1]. However, only a few provincial evaluations have been publicly published at the moment: two of which, namely Liaoning and Anhui Province, reported that neither of them achieved the mid-term goal [14, 15]. Therefore, it is essential to conduct the latest evaluation to find out whether the core measures truly worked, especially in places with serious CS epidemics.

Considering that the evaluation of health measures involves usually real-world research, time series methods would be appropriate for analysis and they have been commonly performed in research about sexually transmitted diseases [16, 17]. Interrupted time series analysis (ITSA) is one of the widely used approaches to assess the effect of health measures introduced at the population level over a clearly defined period [18]. It has been increasingly popular in the fields of PMTCT [19]. In this study, we applied ITSA to detect if there were significant changes in the level and slope in the CS notification rate after implementing the CS control and preventive measures since 2012 to evaluate their effectiveness.

Another time series method, autoregressive integrated moving average models (ARIMA), could overcome the seasonal fluctuation of a linear trend and random error [17]. It has four steps including time series stationary, model identification, parameter estimation and diagnostic checking [20]. We used ARIMA model in this study to forecast CS case numbers after the implementation to assess the future effects of the CS control measures.

Therefore, this study aims to provide the most recent assessment of CS preventive and control measures in Guangdong Province using time series models. We would like to identify whether the measures that Guangdong took were effective, and if so what would be the most worthful part to be promoted to other countries or regions in need.

## Methods

### Study setting

Guangdong Province was selected as a study site, since it was one of the provinces with the highest CS notification rate in the early 2000s. For example, it reported the CS notification rate as 0.45 per 100,000 live births in 2004 and 0.81 per 100,000 live births in 2005, both of which ranked fourth place in China [21]. Having the largest number of migrants in China [22] also added extra pressure to CS control and prevention [23]. We stratified all the 21 cities in Guangdong into three regions based on their geographic location and economic development status, namely, the Pearl River Delta (PRD) region, Eastern and Western wings, and the Northern ecological development zone [24]. Specifically, PRD is the most developed region, in which per capita GDP was almost three times more than that of the Northern zone [25]. This study explored the impact of CS preventive and control measures in Guangdong at both provincial and regional levels so that a more comprehensive evaluation can be provided.

### Study design and data source

The interrupted time series (ITS) design was used to estimate the change in CS notification rate before and after the implementation, which was the year 2012 specifically in this study. Although the “Regulations for the prevention of mother-to-child transmission of AIDS, syphilis and hepatitis B” and “The National Program of Syphilis Control and Prevention 2010–2020”, which contained four main preventive and control measures mentioned before were launched in 2010, the policies need time to be fully implemented at each city or county, and 2012 was also the year when Guangdong Health Commission officially announced the implementation [26, 27]. We therefore identified 2012 as the implementation point.

In line with *Diagnosis for Syphilis,* released by the National Health Commission of China [28], the confirmed CS patient was defined as (a) one whose birth mother was a syphilis patient (b) with characteristic clinical manifestations (c) being positive for laboratory tests (darkfield microscopy/ nucleic acid amplification test or serological tests). Syphilis is categorized into B class infectious disease in China [29] and the confirmed cases, the pathogen carrier or suspected cases, defined by the *Diagnosis for Syphilis *[9], need to all be reported to the local health sector within 12 h in urban areas and 24 h in rural areas [30]. Data about CS which contained annual and municipal case numbers from 2005 to 2020 was derived from the provincial case-based surveillance system, maintained by the Guangdong Provincial Center for Skin Disease and STI Control. However, we were unable to obtain the monthly CS notification rate due to the inaccessibility of the monthly number of live births. Only the local confirmed cases were included in this study. The data of the annual live births were generated from Guangdong Health Statistical Yearbook. The information about economic development was obtained from Guangdong Statistic Book.

### Outcomes

The main outcome of this study was the CS notification rate per 100,000 live births, before and after the implementation of CS preventive and control measures.

### Statistical analysis

#### The interrupted times series analysis

The ITSA was performed to detect changes in the slope and level of CS notification rate after releasing the measures mentioned previously. A significant difference in changes between the pre-implementation period (2005–2011) and the post-implementation period (2012–2020) will indicate a significant impact of measures implemented [31]

ITSA requires the linearity and the non-autocorrelation of the data. The former was confirmed by Cochran–Armitage test (*P* < 0.001) and the first-order autocorrelation was detected by conducting Cumby–Huizinga test, then corrected by Newey–West regression (*P* > 0.05). Variables included in the models were a) *time,* ranging from 1 to 16, representing the year 2005–2020; (b) *intervention*, a dummy variable, which was coded as 0 before the intervention and 1 after the intervention; (c) *post-time*, the time count variable after the intervention. It was coded as 0 before intervention and the first observation after intervention and as 1 starting from the second observation afterward. *ε*_*t*_ is the residual at time t, which represents the variation that is not explained by the regression model. The segmented regression model is specified below:

$$Y_{t} = \beta_{0} + \beta_{1} * \, time + \beta_{2} * \, intervention + \beta_{3} * \, post \, time + \varepsilon_{t} ,$$

where *Y*_*t*_ is the CS notification rate, *β*_*0*_ represents the CS epidemic level at the time when the first observation was obtained (2005); *β*_*1*_ represents the slope estimate of CS notification rate number before implementing the intervention, while *β*_*3*_ represents the slope change before and after the implementation; *β*_*2*_ represents the level change of CS notification rate after the intervention took place*.*

#### ARIMA model

ARIMA model has good prediction accuracy for various infectious diseases including syphilis [32], hence it was applied here to forecast the CS epidemic trend in 2021. The stationary sequence is the precondition of ARIMA analysis; however, as the monthly notification rate was not available in this study and the annual values were too few to establish the model, the monthly case number was used instead. The stationary was then validated by the augmented Dickey–Fuller test (*P* < 0.05) after the first-order log differential. The data from 2005 to 2019 was used to construct the model and that of 2020 was for the verification. The general form of the model is ARIMA (*p*, *d*, *q*) × (*P*, *D*, *Q*) *s*, where *p*, *d*, *q* and *P*, *D*, *Q* represent the order of the autoregressive, integrated, moving average parts of non-seasonal and seasonal components, respectively, and s represents the length of the seasonal period (*s* = 12 for 12 months in this study) [20]. These parameters were estimated by the autocorrelation function (ACF) graph and partial autocorrelation (PACF) graph (Supplementary Fig. 3) [17] and chosen by the minimum Akaike’s information criterion (AIC). Coefficients were calculated using the maximum likelihood estimation method (MLE). A white noise sequence of residuals was confirmed by the Box–Ljung test (*P* > 0.05). Diagnostic checking parameters, including the mean absolute error (MAE), root mean square error (RMSE), and mean absolute percentage error (MAPE) were the indicators to evaluate the goodness of fit of the model (Supplementary Table 2).

The ITSA was performed in STATA Ver 15.1 (Stata Crop 2011) and the optimal ARIMA model was selected using forecast::auto.arima() function in R software version 4.1.0 (R project for statistical computing, Vienna, Austria). The test for the regression coefficient was two sided, *α* = 0.05. The map of CS notification rate distribution was drawn by ArcGIS 10.8 (Environmental Systems Research Institute Inc, Redlands, CA, USA.)

## Results

### CS epidemic analysis

A total of 12,687 confirmed CS cases were reported from 2005 to 2020 in Guangdong Province. The increasing trend of notification rate remained until 2007 when it began to decline slightly before reaching the peak in 2011 (128.55 cases per 100,000 live births). It kept decreasing afterward; the lowest notification rate was met in 2020 (5.76 cases per 100,000 live births) and a 94.65% decrease was realized since 2012 (107.76 cases per 100,000 live births) when the measures were implemented. Table1 depicts the CS notification rate in Guangdong Province during the whole duration.

**Table 1:** The CS case number and notification rate of Guangdong Province, 2005–2020

Year	Number of live births	Number of confirmed cases	Notification rate, 1/100,000 live births
2005	826,754	601	72.69
2006	856,062	953	111.32
2007	933,180	1,152	123.45
2008	1076,485	1,182	109.80
2009	1,104,865	1,203	108.88
2010	1,160,645	1,301	112.09
2011	1,209,687	1,555	128.55
2012	1,365,005	1,471	107.76
2013	1,335,902	1,056	79.05
2014	1,279,995	834	65.16
2015	1,285,983	535	41.60
2016	1,328,820	322	24.23
2017	1,528,465	204	13.35
2018	1,317,907	140	10.62
2019	1,292,526	96	7.43
2020	1,406,854	81	5.76

Notification rate = number of confirmed cases/numbers of live births*10,000

**Fig. 1:**
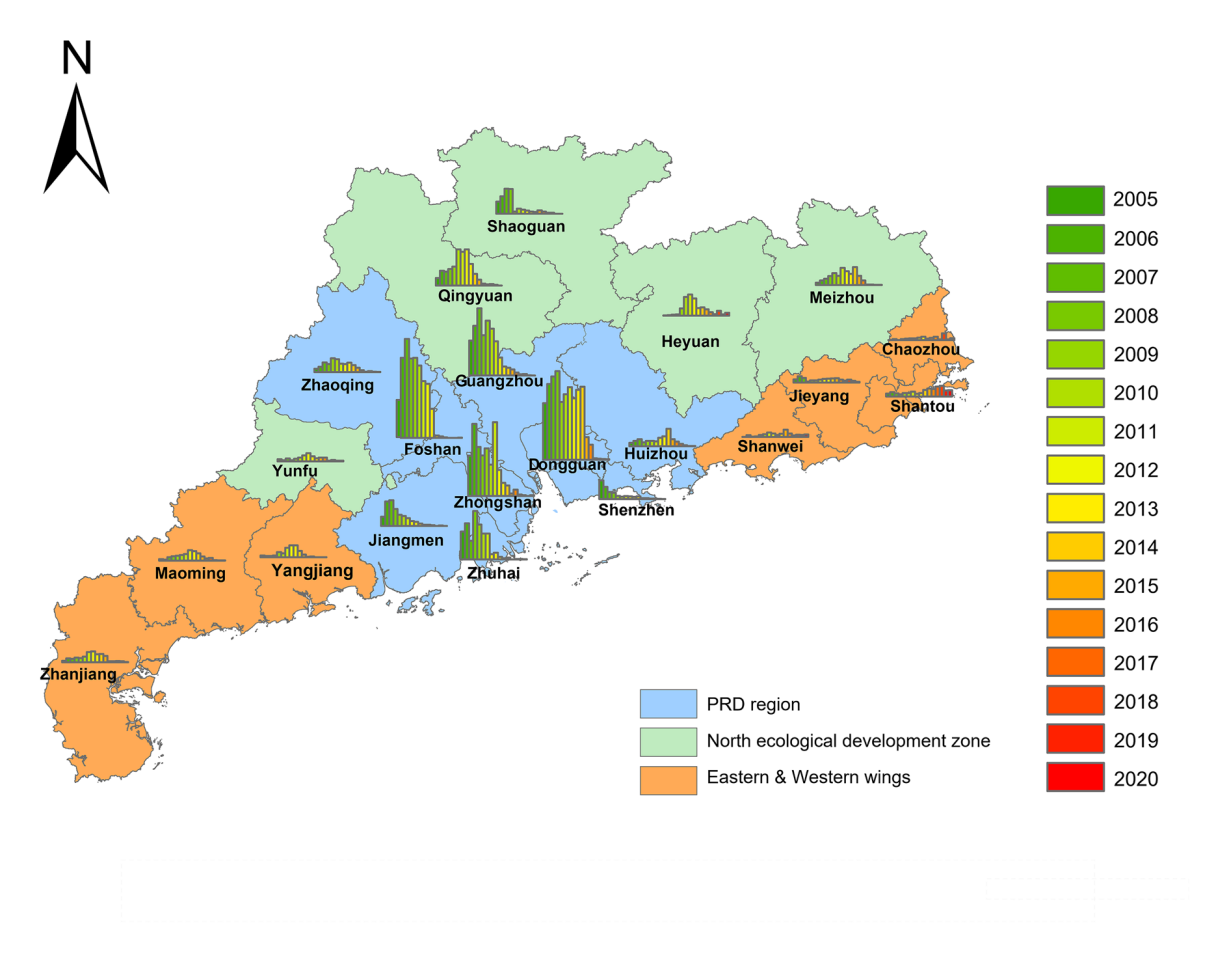
The geographical distribution of congenital syphilis notification rate in each city of Guangdong Province from 2005 to 2020. Blue color represents Pearl River Delta region, green color represents the North ecological development zone and orange color represents the Eastern and Western wings

We explored the geographic distribution of CS notification rate from 2005 to 2020 (See Fig. 1). The notification rate in most cities rose from 2005 and reached the peak around 2012, after which the case number started to fall, coinciding with the implementation period.

**Fig. 2:**
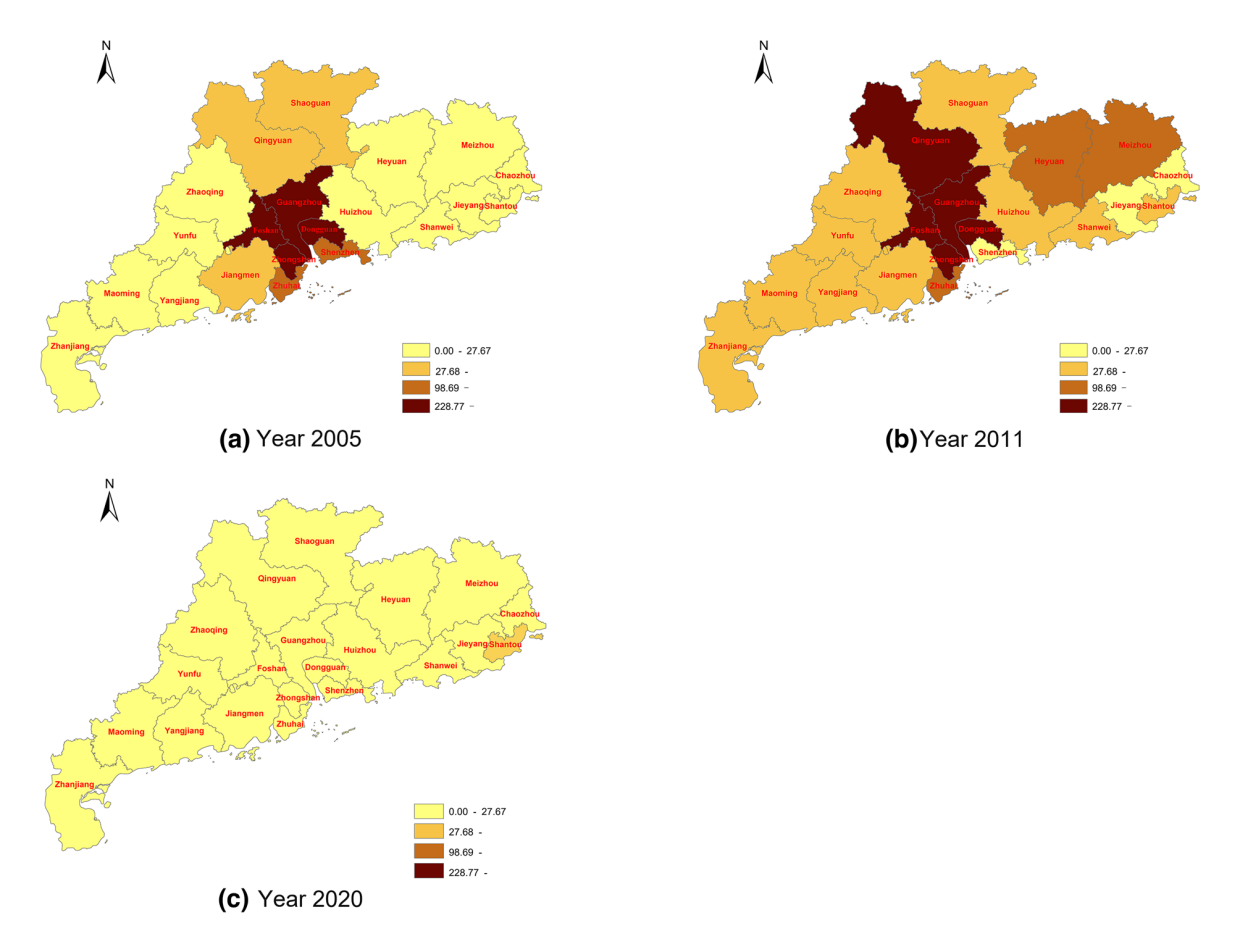
The geographical distribution of CS notification rate in Guangdong Province in 2005 (**a**), 2011 (**b**) and 2020 (**c**). The darker color indicates a higher notification rate

Figure 2 indicates that the CS notification rate mostly clustered at the PRD region in 2005 and then spread to the North ecological development zone and the Eastern and Western wings before the implementation (2011) when the notification rate was generally higher than in 2005. In 2020, most of the cities witnessed a general decrease in CS notification rate, lower than 27.67 cases per 100,000 live births, compared with the other 2 years.

**Fig. 3:**
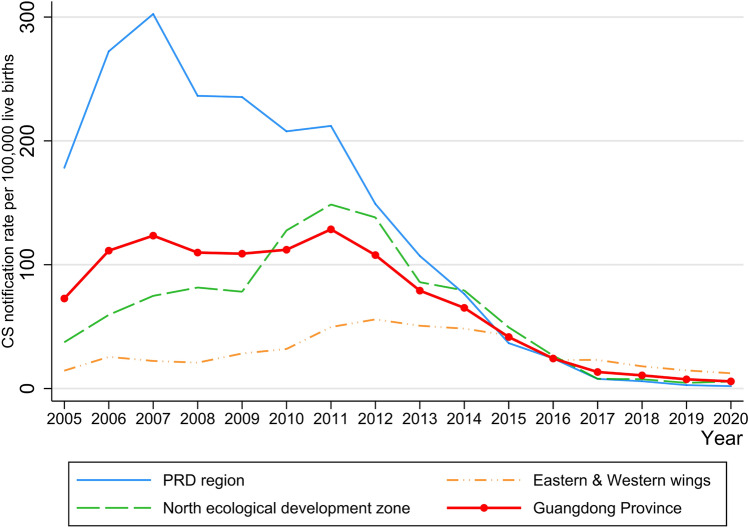
The annual congenital syphilis notification rate of Guangdong Province and three regions from 2005 to 2020

Specifically, the CS notification rate in the PRD region has been decreasing since 2007, while that of the other two regions had a slight increase, peaking around 2011–2012 (See Fig. 3). The distinctive difference in CS notification rate among these three regions can be observed, indicating that the PRD region has achieved the lowest CS notification rate among all the regions since 2015.

### The effectiveness of CS preventive and control measures

**Fig. 4:**
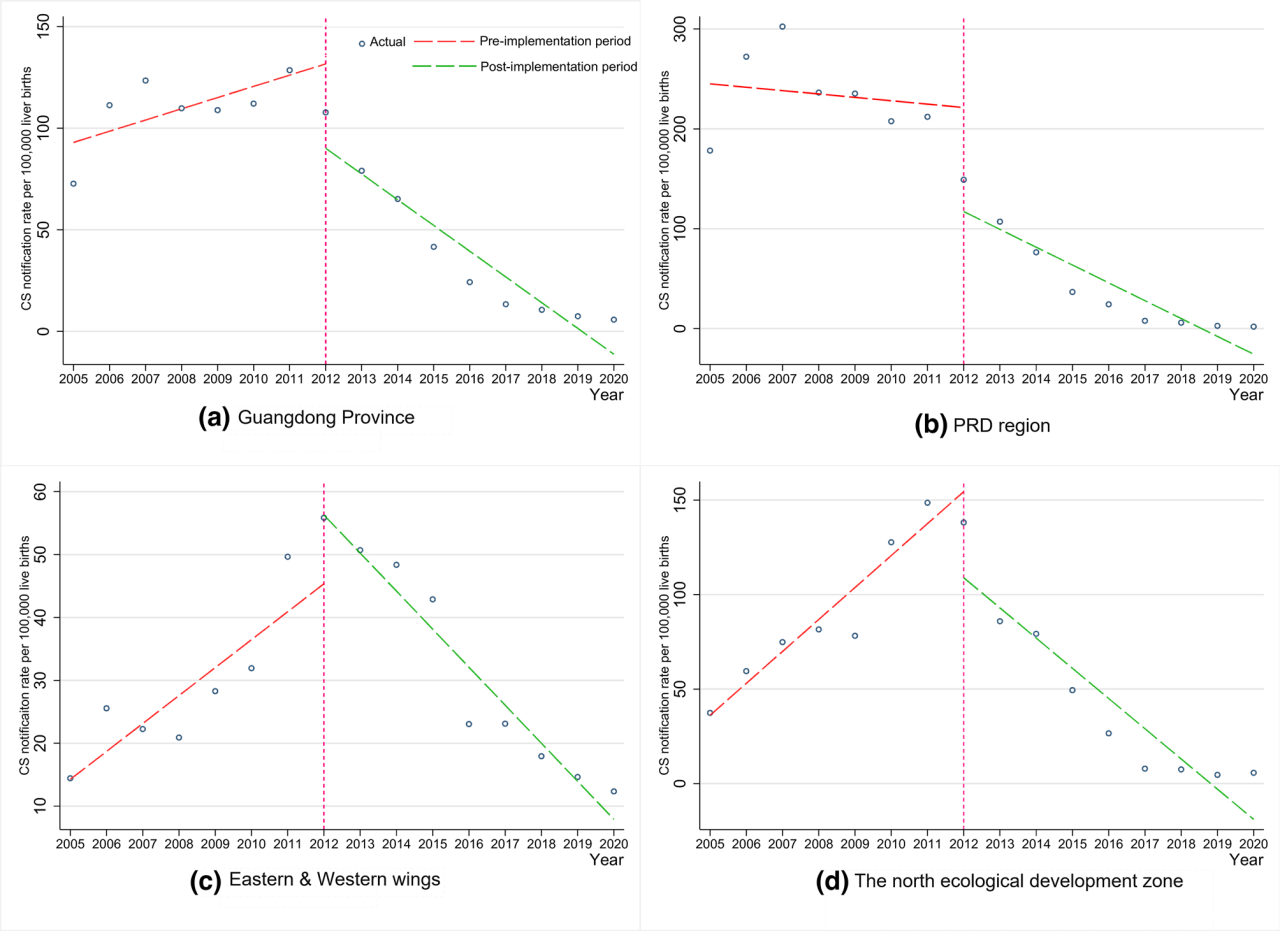
Interrupted time series plot of annual change of congenital syphilis notification rate in Guangdong Province and the three regions before and after the implementation of the CS control measures. The intervention year was 2012. The slope and level change of the province were both significant, *P* < 0.05 (**a**). The level change in the PRD region (**b**) and North ecological development zone (**d**) were both significant, *P* < 0.05; the slope change in the North ecological development zone (**Fig. 4d**) and Eastern and Western wings (**c**) were both significant, *P* < 0.05

Table 2 displays the parameter estimation results of the interrupted time series analysis of Guangdong Province and all three regions. Based on it, we can find that the level change of CS notification rate of Guangdong Province when the measures were implemented showed a statistical significance (*β*_2_ = − 41.50, 95% CI − 67.14 to − 15.85, *P* = 0.004). The slope change of CS notification rate was − 18.19 (*β*_3_ = − 18.19, 95% CI − 25.63 to − 10.75, *P* < 0.001), implying a significant declining trend. In terms of the three regions, the level change of notification rate was statistically significant (*P* < 0.05) in the PRD region and the North ecological development zone (See Table 2). The obvious change of slope was only found in non-PRD regions, which both showed an increasing pre-implementation trend (2005–2011) and turned out to be declining in the post-implementation period (2011–2020) (see Fig. 4).

**Table 2:** Results of multiple linear regression parameter estimations

Variables	Coefficient	Standard error	*t*	95% CI	*P*
Guangdong Province					
Intercept (*β*_0_)	87.46	14.77	5.92	55.29 to 119.64	< 0.001
Time (*β*_1_)	5.52	2.62	2.11	− 0.19 to 11.23	0.057
Intervention (*β*_2_)	− 41.50	11.77	− 3.52	− 67.14 to − 15.85	0.004
Post-time (*β*_3_)	− 18.18	3.41	− 5.33	− 25.62 to − 10.75	< 0.001
PRD region					
Intercept (*β*_0_)	248.47	48.18	5.16	143.50 to 353.45	< 0.001
Time (*β*_1_)	− 3.38	8.67	− 0.39	− 22.28 to 15.51	0.703
Intervention (*β*_2_)	− 104.22	33.13	− 3.15	− 176.39 to − 32.04	0.008
Post-time (*β*_3_)	− 14.47	48.18	− 1.57	− 34.60 to 5.65	0.143
Eastern and Western wings					
Intercept (*β*_0_)	9.81	4.40	2.23	0.22 to 19.39	0.046
Time (*β*_1_)	4.44	1.14	3.89	1.95 to 6.93	0.002
Intervention (*β*_2_)	10.94	6.29	1.74	− 2.76 to 24.65	0.107
Post-time (*β*_3_)	− 10.49	1.21	− 8.67	− 13.13 to − 7.86	< 0.001
North ecological development zone					
Intercept (*β*_0_)	19.19	6.31	3.04	5.43 to 32.94	0.010
Time (*β*_1_)	16.91	2.01	8.40	12.52 to 21.29	< 0.001
Intervention (*β*_2_)	− 45.54	15.07	− 3.02	− 78.39 to − 12.68	0.011
Post-time (*β*_3_)	− 32.88	4.03	− 8.15	− 41.67 to − 24.09	< 0.001

**Fig. 5:**
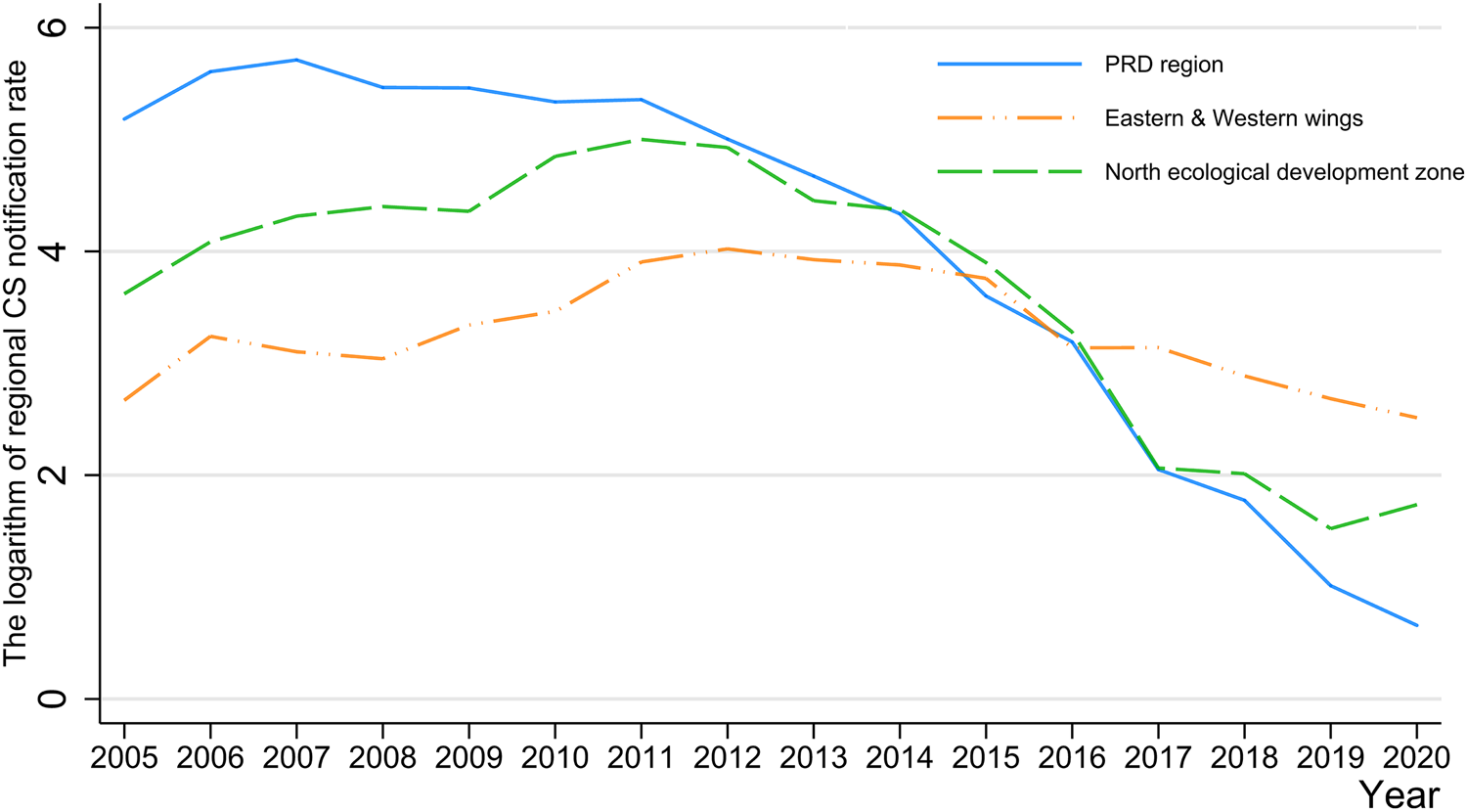
The semi-logarithm line graph of the regional congenital syphilis notification rate from 2005 to 2019

Considering that the values of CS notification rate among the three regions varied greatly, a semi-logarithmic line graph (Fig. 5) was made to compare the rate at which the CS notification rate declined over time. It demonstrated that the notification rate of the PRD region dropped faster after 2012 (the implementation year), compared with the pre-implementation period and showed the fastest declining rate especially since 2017. Hence, although no significance was observed in the PRD region in ITSA, Fig. 5 illustrates that measures implemented since 2012 have helped the PRD region to accelerate the decline in the CS notification rate. The Eastern and Western wings had a relatively stable trend, instead of a downward trend, which explained the insignificance of its level change. The Northern ecological development zone presented an obvious faster drop rate since 2012. The results of both these regions supported that of ITSA.

### The forecasting of CS case number in Guangdong Province

**Fig. 6:**
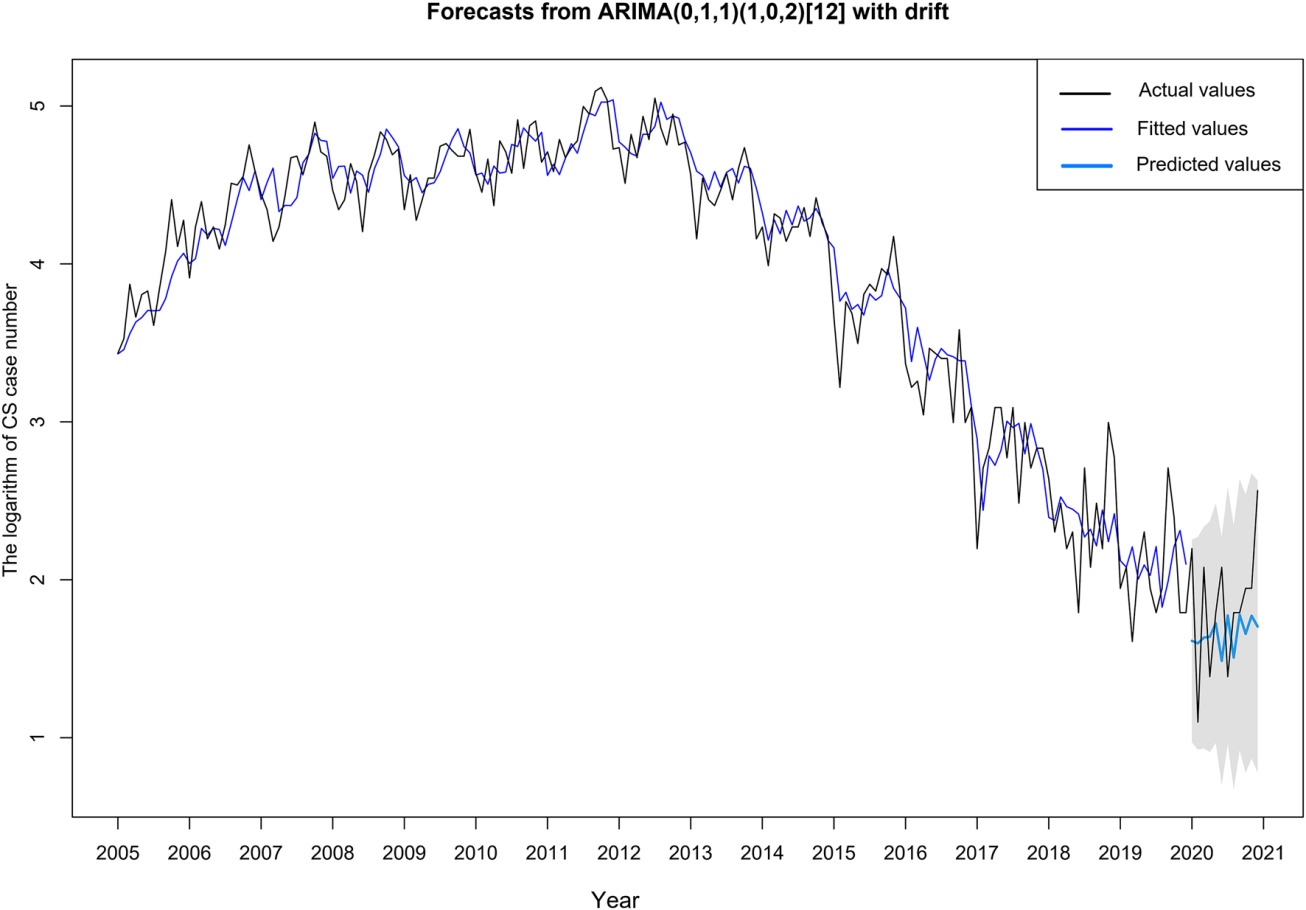
Monthly CS case number from 2005 to 2019 as reported (in black) and fitted by the optimal ARIMA model (in blue). ARIMA-based forecast of CS case number was presented in sky-blue color and displayed a 95% prediction interval (gray ribbon)

Based on the monthly CS case number, the time sequence diagram is displayed in Supplementary Information. In line with the diagram, the monthly CS case number from 2005 to 2020 was not stationary and the first-order log differential was thus performed. The optimal model ARIMA (0,1,1) × (1,0,2) [12] was selected based on AIC and its prediction accuracy was confirmed by a white noise residual sequence (Box–Ljung test: *P* > 0.05). The model presented a satisfying fitting result (See Fig. 6) and predicted the CS case number in 2021 as 48, of which the monthly case numbers are all located within 95% CI. The forecasting result is presented in Supplementary Table 3.

## Discussion

Several policies with a bunch of CS preventive and control measures were launched during the same period (2012–2020), which involved integrating the syphilis screening with health education, the standard treatment for pregnant women and the follow-up of infants of CS patients, alongside supportive strategies from the government. These policies targeted at eliminating MTCT of syphilis and turned out to be effective in Guangdong Province, as evidenced by the significant difference in CS notification rate before and after the implementation.

Particularly, at the provincial level, the notification rate of CS rose from 72.69 in 2005 to 128.55 cases per 100,000 live births in 2011, which was the pre-implementation period, reflecting the heavy disease burden that the re-emergence brought. After implementing the CS control measures, Guangdong Province realized a 94.65% decline in the CS notification rate from 2012 to 2020. This significant change demonstrated the effectiveness that can be attributed to the CS preventive and control measures. However, the severity of the CS epidemic has spread from the coastal region (PRD region) to the inland regions before 2011, which is consistent with the national-wide trend [33]. All the regions gradually had CS controlled under a low-level epidemic after the implementation and achieved the goal set for 2025 in 2017, earlier than planned (2020). Moreover, the CS cases are predicted to be few in 2021. These all indicated that the measures they took earlier were sustainably effective and long-term rewarding.

In terms of regional level, two less-developed regions (Eastern and Western wings and North ecological development zone) both had a rising trend of CS notification rate before the implementation. This could presumably bring a heavier disease burden, as they possessed fewer medical resources and poorer public health capacity than PRD region. In line with Guangdong Province Statistic Bureau [34], the North ecological development zone had the lowest GDP and pregnancy screening rate and the fewest hospitals, but the highest perinatal mortality rate among all the three regions, followed by the Eastern and Western wings. Additionally, recent research found that pregnant women in underdeveloped regions had a higher risk of contracting syphilis. This was because their husbands, who might be ‘return migrants’ from urban to rural areas, possibly had untreated syphilis or high-risk sexual behaviors due to the lack of awareness of the disease. It was thus likely that they transmitted the disease to their wife, leading to the occurrence of CS [35]. In this study, the North ecological development zone had the highest population mobility [36], implying a larger number of migrant returnees. This should have explained why its CS notification rate was higher than the Eastern and Western wings, where the population mobility was less active. Low- and middle-income countries (LMICs) such as Tanzania [37], Mozambique[38] and Nepal [39] have also identified this phenomenon as a potential source of additional CS cases. Moreover, the health professional, as well as the public had poorer awareness of syphilis-related knowledge, compared with those of the PRD region. This would cause missed diagnosis or delayed treatment, implying more cases [40].

All the above suggested the weak capacity and the urgency to support them in preventing and controlling CS from various aspects. However, the supportive measures launched in 2012 have overcome these disadvantages and ensured a successful implementation of other interventions. These measures involved improving the surveillance system, assuring financial subsidy, strengthening the capacity building and so on. For example, regular supervision was conducted following the order of “provincial–municipal–county level” and the central government would allocate particular subsidies to CS control every year based on the number of people in need [41]. All above were referred to as “governance commitment” that was called for by WHO [1]. The situation in both underdeveloped regions in this study started to improve when these measures were launched. Yingde is a vivid example, a county-level city under the jurisdiction of Qingyuan City that locates in the North ecological development zone, with a population of 1.19 million. Before the implementation, Yingde City had only one standard STD laboratory [42]. They then established 15 STD laboratories and 21 syphilis screening laboratories during the post-implementation period, attributed to conducting 10-year national syphilis control plans [43]. This enabled them to conduct large-scale screening. The syphilis screening rate rose from 34.32% in 2013 to 112.74% in 2018 [43]. Together with interventions targeting the high-risk population, the CS notification rate in Yingde City decreased from 4.70 per 100,000 live births to 0.08 per 100,000 live births from 2012 to 2019, which is the post-implementation period [43]. The research conducted in Shanwei City located in the Eastern wings also proved that it was the comprehensive implementation of preventive and control measures that led to a low-level CS epidemic during recent years [44].

PRD, the region with the highest GDP and the most severe CS epidemic before the implementation [34], witnessed a clear decreasing trend since 2007 and soon became the region with the lowest CS notification rate. It was a consensus decades ago that the developed regions tended to suffer a heavier disease burden of CS [45] and urban citizens were more vulnerable than rural ones because of their more frequent high-risk sexual behaviors [46]. Despite this, owning over 80% of GDP [47] and 70% of top-tier hospitals [48] over the province brings the PRD region abundant medical resources and the great capacity to prevent and control the disease. For instance, Shenzhen, one of the most developed cities in the province, launched the *Program of Prevention of Mother-to-Child Transmission of Syphilis in Shenzhen* (PPSS) in 2002, which involved free syphilis tests for pregnant women, treatment for positive individuals and 18 months’ follow-up for their babies [49], which were similar to measures mentioned in this study. The program could cost almost $637 thousand US dollars only in one single year [50], resulting in a remarkable decrease in CS notification rate after 10-year implementation, from 115.3 cases per 100,000 live births in 2002 to 10.4 cases per 100,000 live births in 2011[49].

Due to its strong economy, Shenzhen City had the capacity to implement CS control and prevention measures (2002) before the whole province (2012). It then realized the decrease of CS notification rate earlier than other cities in the PRD region, based on our finding. Hence, we can conclude that as long as supportive measures such as financial subsidy can be well guaranteed, the other interventions on CS control will be always work on the track and bring great benefits. As for underdeveloped regions, although China’s 10-year national syphilis control program pointed out that it should be the local or central government that conducted the supportive measures, they also encouraged the cooperation with international organizations to strengthen the feasibility of the implementation. Therefore, WHO, World Bank, etc., could be considered to provide support such as funding and personnel training for countries in need.

CS is still endemic in many resource-limited regions, which account for most cases globally [1]. These countries might face similar challenges with two underdeveloped regions in our study, such as insufficient medical resources, the weak public health capacity and a large number of migrants, as previously mentioned. WHO [1] pointed out that there was a lack of guidelines for health service providers in resource-limited countries to prevent this disease, and the experience of Guangdong Province suggested the necessity to meet the public health need by providing supportive measures so that other evidence-based interventions can work smoothly.

The generalization of these measures would also be considered as part of international cooperation, which is of great importance for global CS elimination. Additionally, more problems would be also found out when localizing, which can help them to remove obstacles and get closer to the CS elimination eventually.

## Limitations

Several limitations existed in this study. First, more than 18 observations were usually recommended for ITSA [51]. However, the data before 2005 in this study were not obtained because of the upgrade of the national reporting system and we thus have only 16 observations. Despite this, multiple factors such as the sample size per time point and location of intervention in the time series also need to be taken into consideration when applying ITSA [52]. The sample size per time point of this research came from over 12,000 CS cases and the intervention point was located right in the middle of the whole duration. These could both increase the power of ITSA, which compensated for the impact of insufficient observations [52]. Second, the effects of confounding factors such as population mobility could not be examined because of the limited information. Further research at the individual level is therefore suggested. Finally, this study only analyzed the data of Guangdong Province; therefore, more consideration will be required when making the general conclusion about the effectiveness of these measures in other contexts.

## Conclusions

The preventive and control measures derived from two national policies, both focusing on CS have greatly contributed to controlling the epidemic in Guangdong Province, which has achieved the goal for 2025 earlier than scheduled. As a part of these comprehensive measures, the supportive ones could ensure the feasibility and effectiveness of the others. For countries that still have an upward CS epidemic trend but with constrained resources, a satisfying outcome could be expected if support in finance, professional training and public health capacity building and so on can be offered. In that case, the world could move closer to WHO’s goal of eliminating congenital syphilis by 2030.

## Supplementary Information

Below is the link to the electronic supplementary material.

Supplementary file1 (PDF 381 kb)

## Data Availability

The dataset analyzed during the current study is not publicly available, as it was derived from the national system and only staff with permission have the assessment. If data is truly needed, please contact the corresponding author Wang Cheng
