# Supplementary material for: The evaluation of preventive and control measures on congenital syphilis in Guangdong Province, China: a time series modeling study
Source: Infection. 2022 Mar 17;50(5):1179–90. doi: 10.1007/s15010-022-01791-1 (PMC9522686; doi:10.1007/s15010-022-01791-1)
Supplement: Supplementary file 1 — Supplementary file1 (PDF 381 kb) [file 15010_2022_1791_MOESM1_ESM.pdf]

Supplementary Information

For

The Evaluation of Preventive and Control Measures on Congenital Syphilis in Guangdong Province,China: A Time Series Modelling Study

XiJia Tang[1], MSc; Wen Chen[1], MD; ShangQing Tang[1,4], MSc;

PeiZhen Zhao[2,3], MSc; Li Ling[1], MD; Cheng Wang[2,3], MD.

Affiliations:

1 School of Public Health, Sun Yat-sen University, Guangzhou, 510080, Guangdong, China;

2 Dermatology Hospital, Southern Medical University, Guangzhou, 510091, Guangdong, China;

3 Institute for Global Health and Sexually Transmitted Disease, Southern Medical University, Guangzhou 510091, Guangdong, China.

4 Sun-Yat Sen University Cancer Center, Guangzhou, 510080 Guangdong, China.

Corresponding author:

Cheng Wang; Email address: wangcheng090705@gmail.com; Tel: +86 13602463723

Li Ling; Email address: lingli@mail.sysu.edu.cn; Tel: +86 13600097711;

This file includes:

Fig. S1, Fig. S2, Fig. S3, Table. S1, Table. S2,and Table. S3.

Fig S1. Time sequence diagram of monthly CS case during 2005-2020 in Guangdong Province

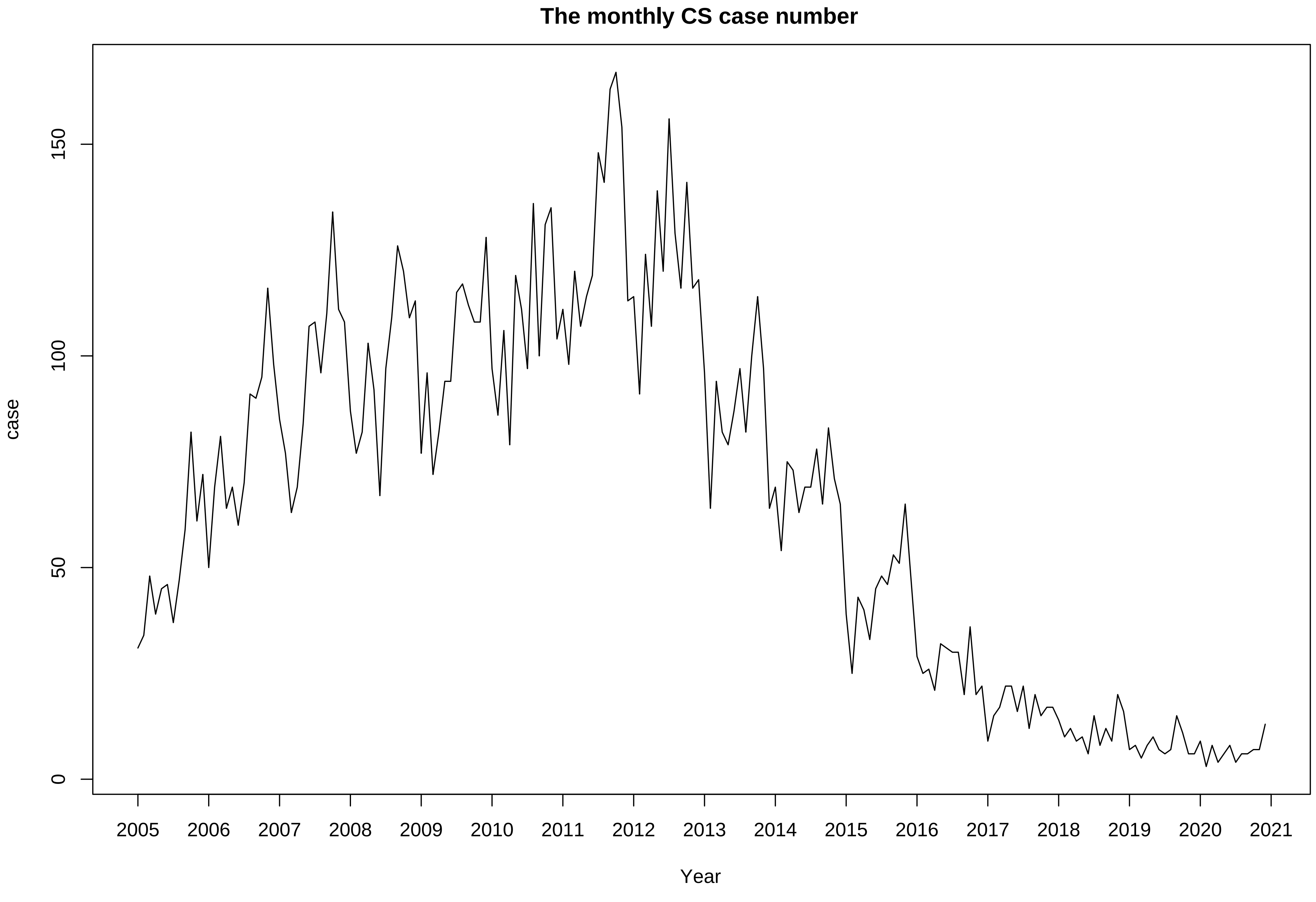

Fig S2. The time sequence diagram of CS case number from 2005-2020 in Guangdong Province after 1st order log differential

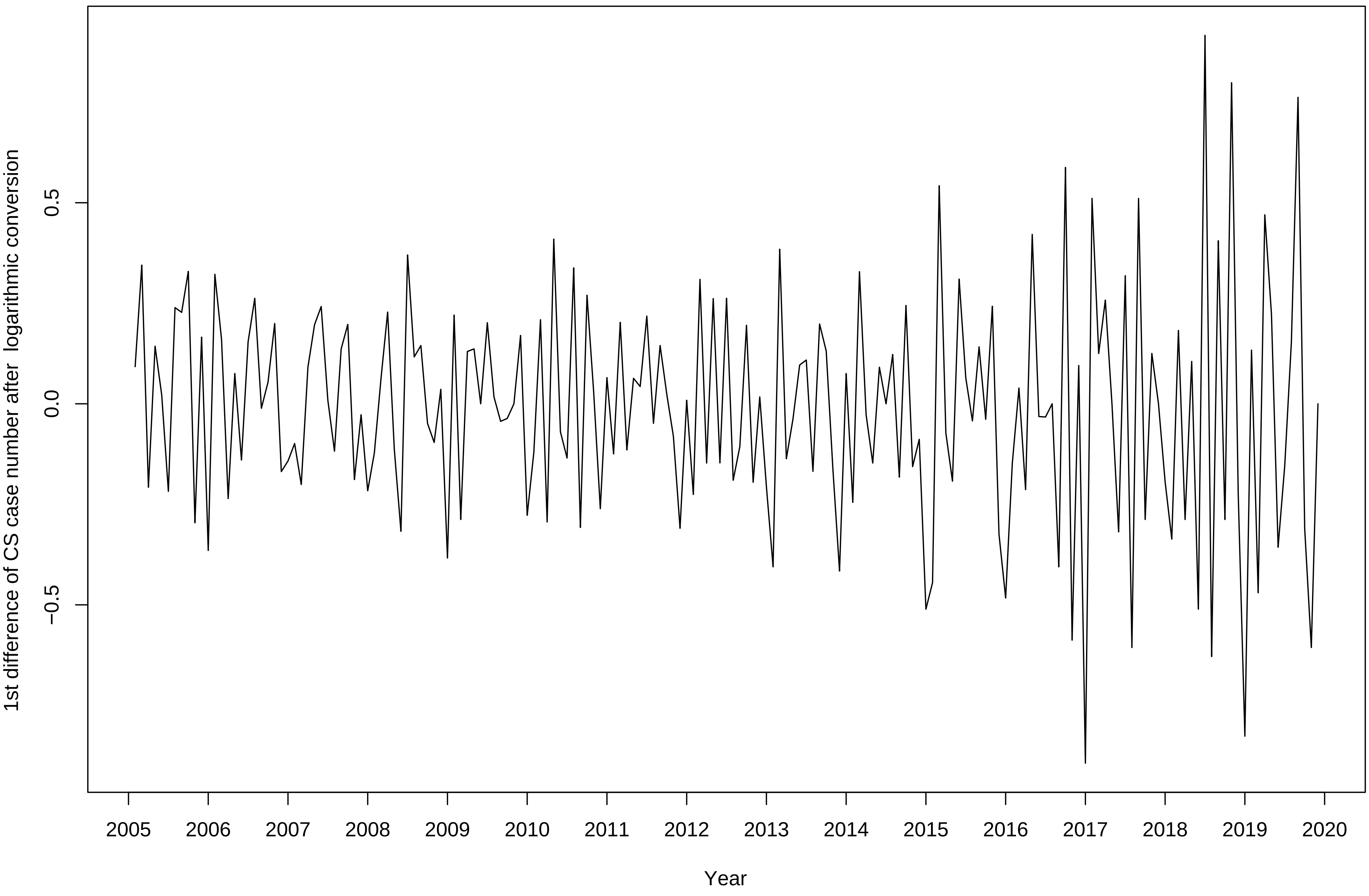

Fig S3. The correlogram graph for CS case number after logarithmic transformation and 1st difference, (a) presents ACF graph and (b) presents PACF graph

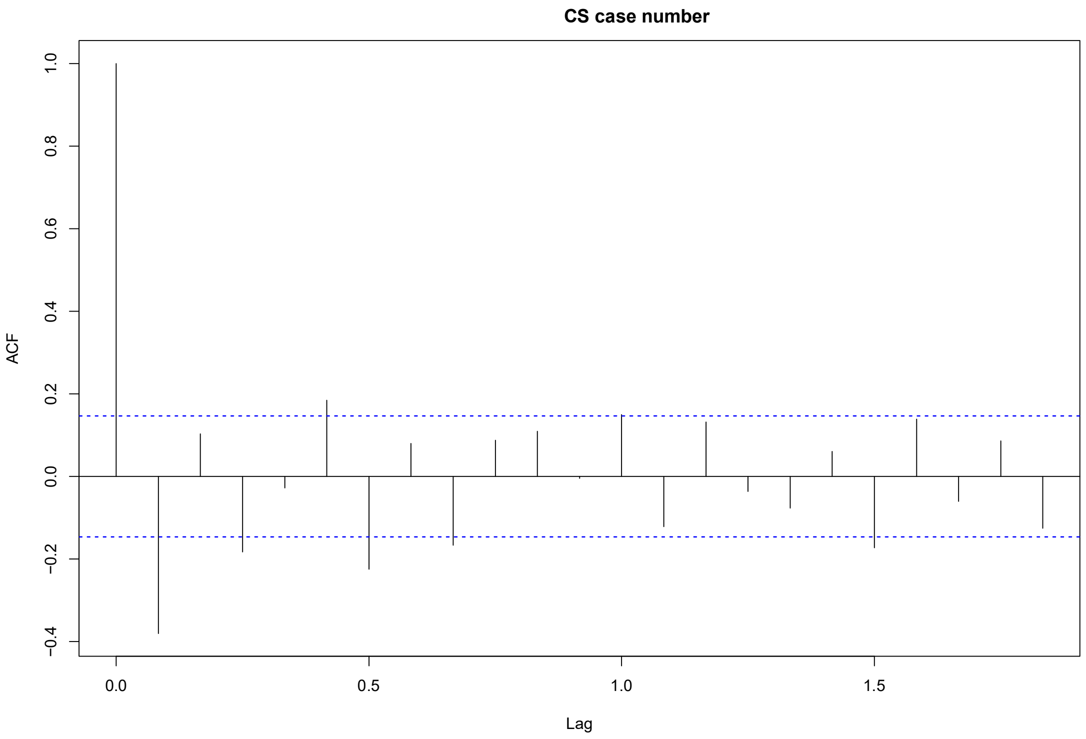

(a)ACF

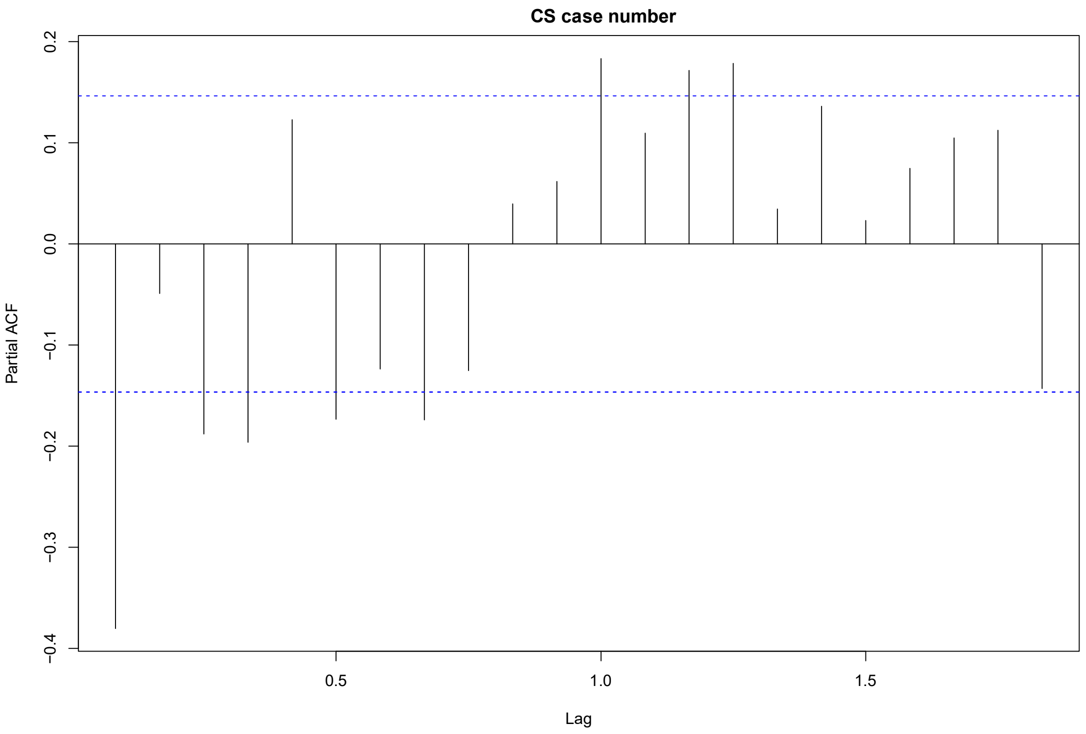

(b)PACF

Table S1. The monthly cases number of congenital syphilis in Guangdong province from 2005-2020

	Year																Total
	2005	2006	2007	2008	2009	2010	2011	2012	2013	2014	2015	2016	2017	2018	2019	2020	
Month																	
1	31	50	85	87	77	97	111	114	96	69	39	29	9	14	7	9	924
2	34	69	77	77	96	86	98	91	64	54	25	25	15	10	8	3	832
3	48	81	63	82	72	106	120	124	94	75	43	26	17	12	5	8	976
4	39	64	69	103	82	79	107	107	82	73	40	21	22	9	8	4	909
5	45	69	84	92	94	119	114	139	79	63	33	32	22	10	10	6	1011
6	46	60	107	67	94	111	119	120	87	69	45	31	16	6	7	8	993
7	37	70	108	97	115	97	148	156	97	69	48	30	22	15	6	4	1119
8	47	91	96	109	117	136	141	129	82	78	46	30	12	8	7	6	1135
9	59	90	110	126	112	100	163	116	100	65	53	20	20	12	15	6	1167
10	82	95	134	120	108	131	167	141	114	83	51	36	15	9	11	7	1304
11	61	116	111	109	108	135	154	116	97	71	65	20	17	20	6	7	1213
12	72	98	108	113	128	104	113	118	64	65	47	22	17	16	6	13	1104
Total	601	953	1152	1182	1203	1301	1555	1471	1056	834	535	322	204	141	96	81	12687

Table S2. Goodness-of-fit test and selection of optimal ARIMA model

Time series	Optimal Model	Goodness-of-fit					Ljung-Box test	
		MAE	RMSE	MAPE	AIC	BIC	χ^2	*P*
The monthly CS case number	(0,1,1) × (1,0,2)[12]	0.36	0.43	0.20	-5.29	13.84	0.01	0.93

Table S3. The forecasting result of CS case number in 2021 using ARIMA (0,1,1) (1,0,2) [12] model

Month	The year 2021		
	Predicted case	Upper 95% CI	Lower 95% CI
1	4	1	10
2	4	1	10
3	4	1	10
4	4	1	11
5	4	2	13
6	3	1	10
7	4	1	12
8	3	1	11
9	5	2	16
10	4	1	14
11	4	1	14
12	4	1	13
Total	48	-	-
